# Remarks on the Practice in Cases of Twins

**Published:** 1811-05

**Authors:** 


### *To the Editors of the Medical and Physical Journal.*

### *Remarks on the Practice in Cases of Twins.*

GENTLEMEN,

I HAVE read the case of twins communicated by your Correspondent, Mr. Jones, (page 311) with much interest, and think it valuable, inasmuch as it proves, that under some circumstances the second of twin children may be safely left in the womb for an unusual length of time after the birth of the first. But I cannot admit, that this case at all supports the doctrine of superfetation; nor can I agree with him in the opinion which he wishes to draw from it, "that it would be more prudent, *if no unfavourable symptoms occur*, to leave the expulsion of the second child to nature, rather than to interfere according to the present practice."

Considered simply as a matter of prudence, I conceive, that it is generally right, not to allow the second child to be retained (unless under such unavoidable circumstances as your correspondent mentions) for many hours after the birth of the first. If it were to become an established maxim, that the accoucheur was not to interfere, when the second child is retained in the uterus, *unless unfavourable symptoms should arise*, it is to be apprehended, that his interference would sometimes be delayed, till it would be impossible to interfere with effect, should any alarming symptoms come on.

I know a gentleman in the practice of Midwifery, who among his first patients met with a case of twins. The first child having been born about an hour, he began to think that it would be necessary to accelerate the birth of the second child by art: but being unwilling to trust to his own judgment, he requested the advice of a senior practitioner. The senior gave a very decided opinion, that no assistance was necessary, and that time should be allowed for nature to expel the child. Hour after hour passed away, in waiting for the spontaneous action of the uterus, with much impatience on the part of the patient and her friends, and much anxiety on the part of the young practitioner, till the opportunity of delivering by art was gone by. On the third or fourth day after the birth of the first child, a violent hæmorrhage from the uterus took place, which could by no means be restrained, and the poor woman very soon expired, after having excluded the second child in a lifeless state. This case alone sufficiently proves the danger of leaving the management of twin cases entirely to nature, and this is not the only one attended with fatal consequences from similar delay, which I am acquainted with.

There

There is much analogy between the cases of the second of twin children retained in the uterus, and the retention of the placenta; in either case it may be sometimes safe, but it may not be always prudent, to trust to nature. The late Dr. William Hunter, at one period of his practice, always left the placenta to be expelled by the efforts of the uterus, and he taught his pupils, that it was a *diabolical* operation to introduce the hand into the uterus for the purpose of separating the placenta. His authority had great weight with other practitioners of midwifery, and it became customary to leave the placenta, not only many hours, but even many days, till at length it was expelled naturally. For some time no unfavourable case occurred; all the women in whom the placenta was left to nature did well, and the Doctor was thought to have introduced a great improvement of the art of midwifery. At last however the scene changed, several women died, some with the placenta still adhering to the uterus, and others only lived to expel the placenta in a state of the most dreadful putrefaction: so that Dr. Hunter was compelled to abandon his doctrine, and to admit the necessity of the interference of art in the management of the retained placenta. Since this period, the treatment of these cases has been conducted upon more rational principles, and perhaps admits now of very little improvement.

I have no hesitation in saying, that in the management of twin cases, we ought to be guided by similar principles. It is well to know that our interference may not always be necessary, but our judgment will probably determine us not to let slip a favourable opportunity of relieving our patient from her apprehensions, and preserving her from the chance of danger, by finishing the delivery while we have it in our power.

OBSTETRICUS.

*April* 8, 1811.

